# Testing for disconnection and distance effects on physiological self-recognition within clonal fragments of *Potentilla reptans*

**DOI:** 10.3389/fpls.2015.00215

**Published:** 2015-04-07

**Authors:** Bin J. W. Chen, Peter J. Vermeulen, Heinjo J. During, Niels P. R. Anten

**Affiliations:** ^1^Ecology and Biodiversity, Institute of Environmental Biology, Utrecht UniversityUtrecht, Netherlands; ^2^Centre for Crop Systems Analysis, Department of Plant Sciences, Wageningen UniversityWageningen, Netherlands

**Keywords:** identity recognition, neighbor detection, physiological integration, root competition, self/non-self recognition, severance effect, source–sink relationship, vegetative propagation

## Abstract

Evidence suggests that belowground self-recognition in clonal plants can be disrupted between sister ramets by the loss of connections or long distances within a genet. However, these results may be confounded by severing connections between ramets in the setups. Using *Potentilla reptans*, we examined severance effects in a setup that grew ramet pairs with connections either intact or severed. We showed that severance generally reduced new stolon mass but had no effect on root allocation of ramets. However, it did reduce root mass of younger ramets of the pairs. We also explored evidence for physiological self-recognition with another setup that avoided severing connections by manipulating root interactions between closely connected ramets, between remotely connected ramets and between disconnected ramets within one genet. We found that ramets grown with disconnected neighbors had less new stolon mass, similar root mass but higher root allocation as compared to ramets grown with connected neighbors. There was no difference in ramet growth between closely connected- and remotely connected-neighbor treatments. We suggest that severing connections affects ramet interactions by disrupting their physiological integration. Using the second setup, we provide unbiased evidence for physiological self-recognition, while also suggesting that it can persist over long distances.

## Introduction

One of the fascinating discoveries in plant biology in recent years is that plant roots are able to detect the presence and even recognize the relatedness of their neighbors (reviewed in Chen et al., 2012; Dudley et al., 2013; Depuydt, 2014) independently of their effect on soil nutrient status (Mommer et al., 2010; Padilla et al., 2013; Schmid et al., 2013). In an evolutionary game-theoretical context, it enables plants to over-proliferate roots for a greater capture of shared resources in competition with non-self roots while avoiding unprofitable competition with self roots (e.g., Gersani et al., 2001; O’Brien et al., 2007). Interestingly, responses to non-self roots not only occur between genetically different plants (e.g., Dudley and File, 2007; Biedrzycki et al., 2010; Fang et al., 2013), but also take place between genetically identical while physically independent individuals (e.g., Falik et al., 2003). The latter scenario might be particularly important for clonal plants. Such plants produce vegetative offspring (ramets) connected by stolons or rhizomes (de Kroon and van Groenendael, 1997), and these connections in turn, disintegrate or senesce over time or after disturbance (Hutchings and Mogie, 1990). Thus, within one genet there are likely to be intensive root interactions between closely connected ramets, between remotely connected ramets, and between disconnected ramets.

Recent studies have shown that severing connections between sister ramets grown together in a pot will induce greater root growth at the expense of aboveground performance (e.g., Holzapfel and Alpert, 2003; Gruntman and Novoplansky, 2004; Falik et al., 2006; Roiloa et al., 2014). Their results were interpreted as showing that these connected ramets exhibit self-recognition mediated by physiological processes, which can be disrupted due to the loss of connections (Gruntman and Novoplansky, 2004; Falik et al., 2006). This further indicates that the loss of connections within a clone would lead to an over-proliferation of roots, which might lead to a reduction in clonal offspring. Interestingly, a study of *Buchloe dactyloides* also showed that two ramet-halves originating several nodes apart on the stolon produced more root mass when grown together than two halves originating from the same node (Gruntman and Novoplansky, 2004). From this, the authors suggested that physiological self-recognition can fade with the distance between two units along the clone. However, so far, other studies testing such physiologically based recognition within a genet including disconnection and distance effects are still lacking.

Moreover, the procedure of comparing intact and severed ramet pairs in the above-mentioned studies can be criticized. Severing connections between sister ramets may affect plant growth in more ways than just preventing the transduction of self-identity signals. It disrupts resource and hormone transportation (e.g., Hartnett and Bazzaz, 1983; Alpert, 1996; Hutchings, 1999; van Kleunen and Stuefer, 1999; Alpert et al., 2002; Liu et al., 2007; Semchenko et al., 2007), as well as the potential for division of labor (Roiloa et al., 2014). Thus, experiments employing a sudden severance of connections to create severed pairs may induce effects that are not associated with the loss of self-recognition. Furthermore, each pair always consists of a developmentally younger and an older ramet. This age effect has been included in the research on physiological integration between ramets in response to abiotically (nutrients, light, or water) environmental heterogeneity (e.g., Alpert, 1999; Roiloa and Hutchings, 2013; Wang et al., 2013). Yet, it has been seldom considered in the studies of physiological self/non-self recognition (but see Roiloa et al., 2014). Therefore, there is a need to re-test such recognition using experimental designs that exclude the effects of severing connections, and include the effects of age.

The objectives of our study are (1) to examine the effects of severing connections on ramet growth in a traditional “intact/severed pair” setup; (2) to introduce a novel setup that avoids severance effects to investigate unbiased evidence for physiological self/non-self recognition in clonal plants; and (3) to explore the disconnection and distance effects on physiological self-recognition within a single genet using this novel setup. For the first objective, we conducted an experiment whereby ramet pairs were grown in pots in the traditional way, i.e., their connections were either severed or kept intact. For the second and third objectives, we conducted another experiment whereby ramets, remaining attached to larger clonal fragments, were grown with closely connected ramets, remotely connected ramets, and disconnected ramets. This second experimental setup avoids the severance of connections and keeps ramets integrated with their maternal fragments, which is more in line with the way root interaction may occur in natural vegetation.

Based on the concerns that severing the connection disrupts physiological integration between sister ramets, we hypothesize that in the first experiment:

1. By removing source–sink relationship between younger and older ramets, severance mainly reduces the growth of younger ramets.

According to the suggestion that physiological self-recognition between genetically identical ramets can be disrupted by disconnection with greater root production as a consequence (Holzapfel and Alpert, 2003; Gruntman and Novoplansky, 2004; Falik et al., 2006), we hypothesize that in our second experiment:

2. Ramets have greater root mass when grown with disconnected neighbors than when grown with closely connected neighbors;

Finally, as the transduction of self-signal within a clonal system could be distance limited, physiological self-recognition can be inhibited by a longer connection between two connected ramets growing closely together, with greater root production as a consequence (Gruntman and Novoplansky, 2004), we hypothesize that in our second experiment:

3. Ramets have greater root mass when grown with remotely connected neighbors than when grown with closely connected neighbors.

## Materials and Methods

### Plant Material and Propagation

The experiments were carried out with the stoloniferous perennial species *Potentilla reptans* L. (Rosaceae). Its common habitats include river and lake shores, moderately disturbed pastures, mown grasslands, and road margins (van der Meijden, 2005). The plant produces sympodially growing stolons with rooted rosette-forming ramets on the nodes. Without strong disturbance, the connections (i.e., internodes) between ramets will function throughout one growing season (Stuefer et al., 2002). This species shows highly plastic responses to both local and non-local environmental cues in above- and belowground parts (e.g., Stuefer et al., 1994; He et al., 2011; Wang et al., 2013), suggesting that it is able to locally adjust belowground allocation, making it suitable for our study.

In April 2013, plants from one genotype were propagated in a greenhouse at Wageningen University, Wageningen, The Netherlands. After 2 months, new-grown rootless ramets were individually pinned in pots (1.0 L, with potting soil). Two weeks later, when root systems were initiated, these ramets were severed from the stock plants and designated as mother ramets. They were then propagated for another month. To promote growth, each pot (i.e., each mother ramet) received nutrient solution (7.79 mM NO_3_^-^, 1.1 mM NH_4_^+^, 1.5 mM PO_4_^3-^, 5.11 mM K^+^, 3 mM Ca^2+^, 1.0 mM SO_4_^2-^, 0.87 mM Mg^2+^, and micronutrients) three times (60 ml per occasion) in this period. One month later, when mother ramets were well developed and had produced several maternal stolons bearing rootless daughter ramets without access to soil, these mother ramets were used in the two following experiments.

### Experiment 1: Effects of Severing Connections

This experiment, which was started on July 29, 2013, followed the traditional setup in which root recognition has been studied in the past, whereby connections in ramet pairs are severed (e.g., Holzapfel and Alpert, 2003; Falik et al., 2006). From each mother ramet, we selected maternal stolons that bore eight (9 or 10 in very limited cases) rootless daughter ramets. To standardize for the developmental stages of ramets throughout all replicates, the third (younger) and fourth (older) rootless daughter ramets counting from the distal position (apex) of each selected maternal stolon were chosen, when both of them had three newly formed leaves and had not produced their own stolons.

**FIGURE 1:**
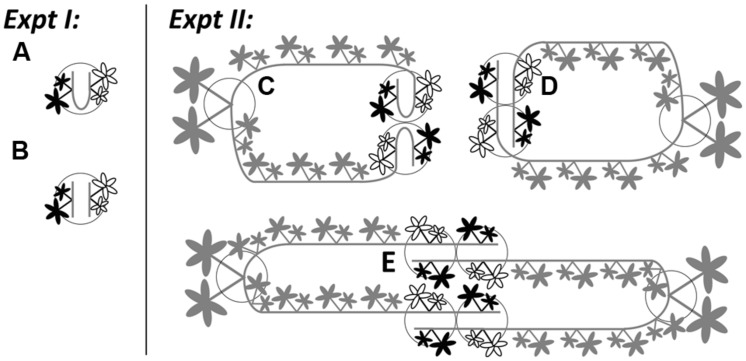
**Illustrations for Experiment 1 (the effects of severing connections) and Experiment 2 (physiological self/non-self recognition) using *Potentilla reptans*.** In Experiment 1, younger (black leaves) and older (white leaves) ramets were grown in **(A)** intact and **(B)** severed pairs. In Experiment 2, younger (black leaves) and older (white leaves) ramets, remaining attached to the maternal stolons of mother ramets, were grown in **(C)** closely connected-, **(D)** remotely connected- and **(E)** disconnected-neighbor treatments. Circles represent pots. Gray parts in Experiment 2 stand for maternal size (including the mother ramet and its two maternal stolons attached with the rest of the daughter ramets). For clarity, the secondary structures of younger and older ramets are not shown.

Then, each chosen pair was pinned and grown in one pot (1.0 L) that was filled with a sand-soil mixture (river sand and sieved nutrient-poor arable soil in 1:1 volume ratio). To increase and also to standardize the extent of root contact, the distance between two ramets within the pair was shortened by bending the internodes between them. One week later, these newly rooted ramet pairs were severed from their maternal stolons. On the same day, for half of these pairs the connections were left intact (**Figure 1A**), while for the other half their connections were severed (**Figure 1B**). Plants were grown under ambient light conditions (*c*. 80% of full sunlight) and watered daily in a plastic-roof-only tunnel, from 5th August to 13th September in 2013. Each treatment consisted of 20 replicates. To promote root competition, no additional nutrients were given in this period. During the experiment, root production on new ramets was prevented.

After 40 days, all younger and older ramets were harvested and divided into roots, leaves, and their newly produced stolons (new stolons hereafter, as noted one stolon is composed of both internodes and attached ramets). During the harvesting, the two root systems within each pair were carefully separated, washed, and assigned to individual ramets (i.e., the younger and the older) together with leaves and new stolons. All materials were weighed after 3 days of oven-drying at a temperature of 70^∘^C. Then, total mass (the summation of root, leaf, and new stolon mass) and root mass fraction (RMF; root mass divided by total mass) was calculated at ramet individual level.

We first examined the effects of severing connections (i.e., intact *versus* severed) on the biomass components of younger and older ramets. Each younger ramet grew in one pot with its older sister ramet, thus the tests were done using linear mixed-effect models in which severance, age and their interaction were fixed effects and pot (or ramet pair) was a random effect. Since growth strategies of plants are generally size-dependent (Coleman et al., 1994; Poorter and Sack, 2012), we tested for the effects of severance on RMF as a function of ramet total mass, on leaf mass, and new stolon mass as a function of root mass in younger and older ramets, using linear mixed-effect models.

### Experiment 2: Physiological Self/Non-Self Recognition

This experiment was started on August 7, 2013. For each mother ramet, we only kept two maternal stolons that bore eight (9 or 10 in very limited cases) rootless daughter ramets (the same standard as in Experiment 1) with all the other maternal stolons removed. To standardize for maternal support (e.g., distance between mother and daughter ramets) in addition to the developmental stages of ramets throughout all replicates, the fifth (older) and sixth (younger) rootless daughter ramets counting from the basal position (mother ramet, opposite to the apex) of each selected maternal stolon were chosen, when both of them had three leaves but had not produced new stolons. As noted, although two selection procedures for younger and older ramets in Experiment 1 and Experiment 2 were based on different selection criteria in view of the different questions being studied, both procedures selected all younger ramets and all older ramets with the same developmental stages, respectively, since we chose all maternal stolons with the same standard (i.e., attached with eight rootless daughter ramets) in both experiments.

Subsequently, we introduced three types of neighbor treatments between genetically identical ramets: (1) closely connected-neighbor treatment, whereby a pair of adjacent daughter ramets from the same maternal stolon were pinned in one pot (**Figure 1C**), representing the control group; (2) remotely connected-neighbor treatment, whereby one younger and one older daughter ramet from different stolons produced by the same mother were pinned in one pot (**Figure 1D**), representing a distance effect; (3) disconnected-neighbor treatment, whereby one younger and one older daughter ramet from different mothers with the same genotype were pinned in one pot (**Figure 1E**), representing a disconnection effect. For the same reason as in Experiment 1, we bent the internodes between ramets in closely connected-neighbor treatment. In addition, before the start of the experiment, the first to fourth daughter ramets on each maternal stolon were left untouched but were not placed in pots to prevent rooting, while daughter ramets distal to the sixth (i.e., the younger one) on each maternal stolon were removed. With this setup, we were able to standardize the internal growth conditions (i.e., physiological integration within one clonal fragment) for all younger and all older ramets throughout the whole experiment. Plants were grown in the same tunnel with the same kind of pots filled with the same type of sand–soil mixture as used in Experiment 1. Each type of treatment consisted of 18 replicates.

After 40 days, all clonal fragments were harvested. During the harvest, younger and older daughter ramets rooted in pots were first marked and severed from the fragments, and were then separated and treated following the same protocol as in Experiment 1. The remaining parts of the clonal fragment, i.e., the mother ramet and its two maternal stolons attached with the rest of the rootless daughter ramets (see **Figure 1C–E**), were collected and assigned together as maternal size. As noted, mother ramets and rootless daughter ramets seldom produced new stolons. If such organs were produced, they were also designated as contributing to maternal size. All materials were weighed after 3 days of oven-drying at a temperature of 70^∘^C.

We first examined the effects of neighbor treatments on the total mass and maternal size of clonal fragments, using linear mixed models with neighbor treatment (i.e., closely connected, remotely connected, and disconnected neighbors) as a fixed effect and treatment unit as a random effect. Subsequently, we tested for the effects of neighbor treatment on biomass components of younger and older ramets as a function of their maternal size, as the growth of a ramet also depends on the size of the clonal fragment to which it is attached (Birch and Hutchings, 1999). Each target ramet grew in one pot with another ramet, but at the same time remained attached to a clonal fragment which included three other target ramets. Thus, the tests were done using linear mixed-effect models with neighbor treatment, age and maternal size and their interactive effects as fixed effects, and clonal fragment and pot (ramet pair) as random effects. We tested for the effect of neighbor treatment on RMF as a function of ramet total mass, and on leaf mass, and new stolon mass as a function of root mass in younger and older ramets, using linear mixed-effect models.

For the analyses of both experiments, the best fitted models were selected using backward selection procedures based on full models, where the Akaike information criterion test was applied for difference in the fit of the nested models (Bozdogan, 1987). Data were transformed when necessary. All statistical analyses were performed using lme4 (Bates et al., 2014) and lmerTest (Kuznetsova et al., 2014) packages in R version 3.1.0 (R Core Team, 2014).

## Results

### Experiment 1: Effects of Severing Connections

**FIGURE 2:**
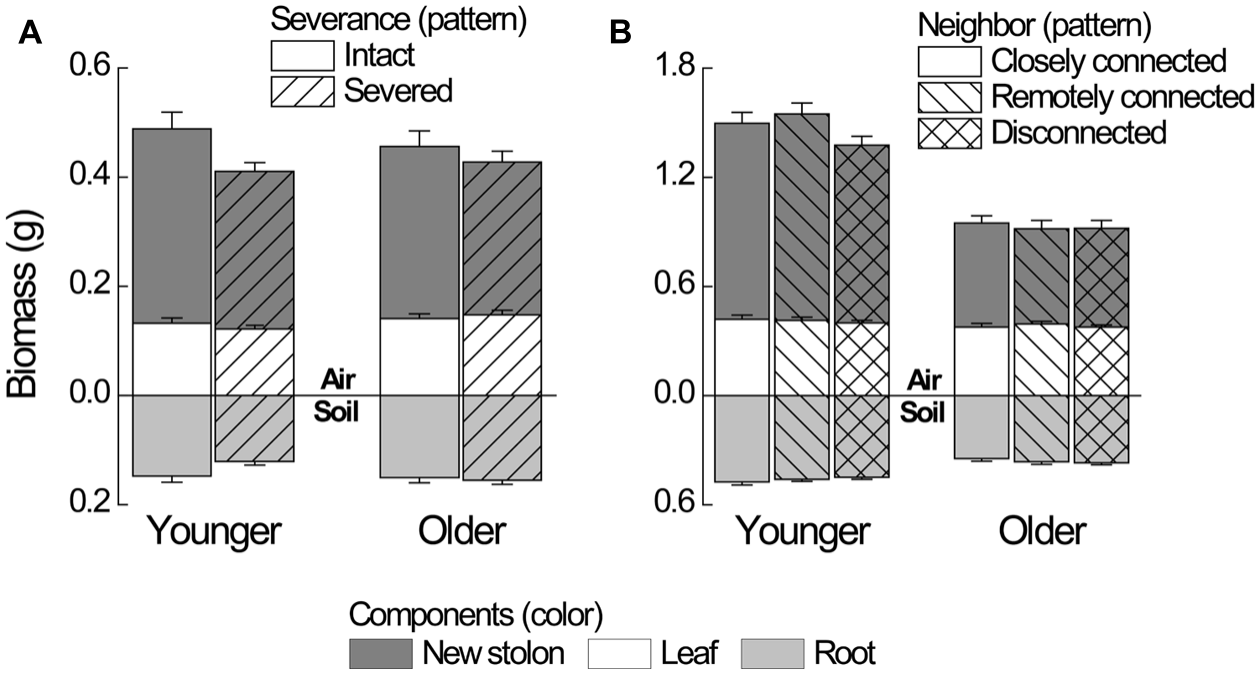
**Biomass components of younger and older *P. reptans* ramets in **(A)** Experiment 1 and in **(B)** Experiment 2.** Error bar denotes 1 SE of the mean.

**FIGURE 3:**
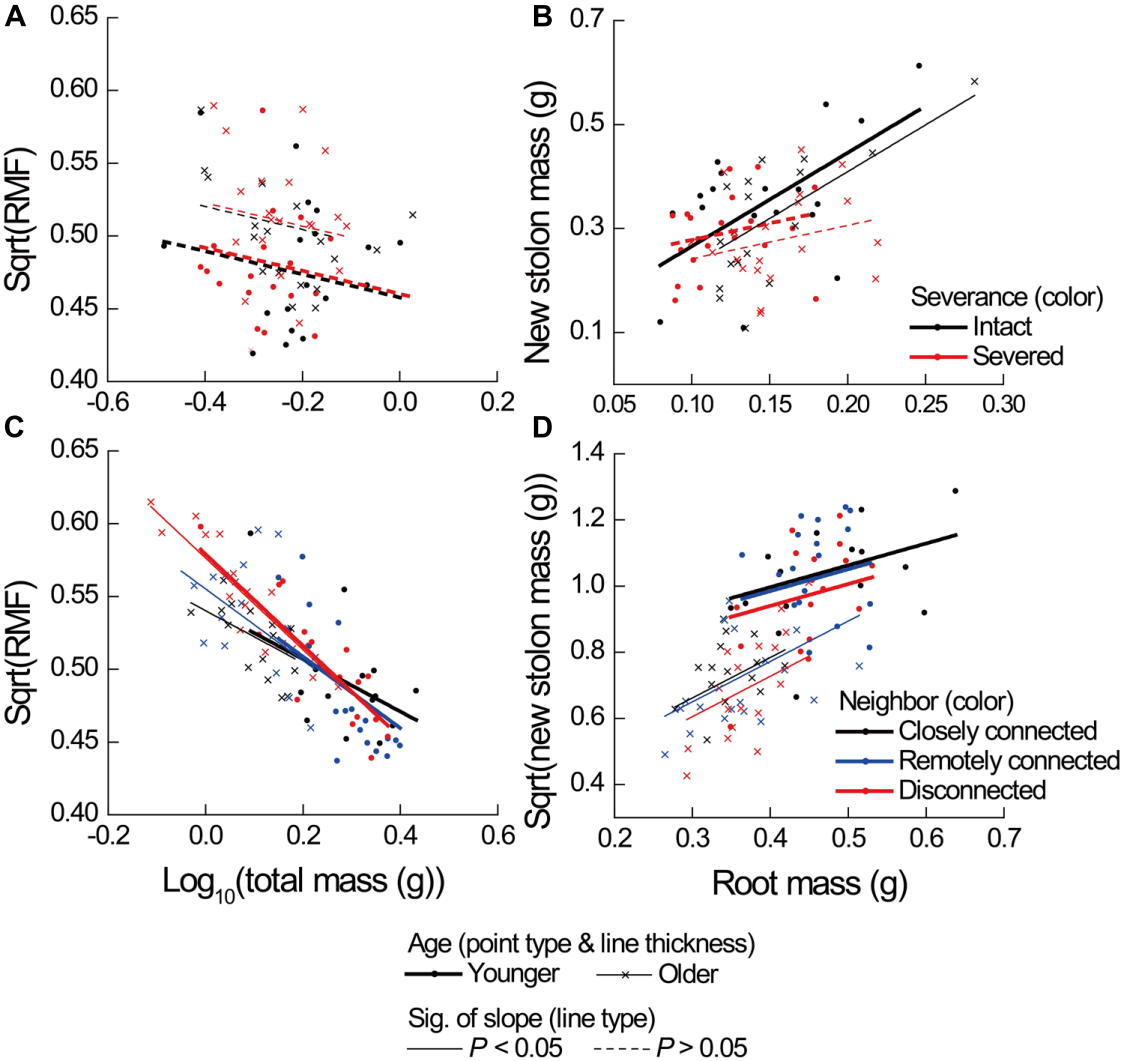
**Effects of severance treatment in **(A,B)** Experiment 1 and neighbor treatment in **(C,D)** Experiment 2 on root mass fraction (RMF) as a function of ramet total mass, and on new stolon mass as a function of root mass of younger and older *P. reptans* ramets.** All regression lines are based on the analyses using linear mixed-effect models (see **Table 2**).

Severance significantly reduced root mass of younger ramets but not that of older ones (**Figure 2A**, significant severance × age in **Table 1A**). However, it had no significant effect on RMF of either younger or older ramets ( **Table 2A**; **Figure 3A**). Both younger and older ramets in intact pairs had their new stolon mass significantly positively (*P* < 0.001) correlated with their root mass, but for those in severed pairs their new stolon mass remained low regardless of their root mass (**Figure 3B**, significant severance × root mass in **Table 2B**). This led to a significantly negative overall effect of severance on the new stolon mass of both younger and older ramets (**Table 1A**). Generally, severance-induced biomass reductions were more pronounced in younger ramets than in older ramets (**Figure 2A**).

**Table 1:** Summary of statistics for the fixed effects on the biomass components of *Potentilla reptans* ramets in Experiments 1 and 2, using linear mixed-effect models.

Fixed effect	Total	Root	Leaf	New stolon
**(A) Experiment 1: The effects of severing connections**
Severance (S)	0.085	0.243	0.825	**0.038**
Age (A)	0.649	**0.014**	**0.026**	0.339
S × A	—	**0.049**	—	—
**(B) Experiment 2: Physiological self/non-self recognition**
Neighbor (N)	**0.026**	0.757	0.688	**0.018**
Age	**<0.001**	**<0.001**	**0.004**	**<0.001**
Maternal size (M)	0.167	**<0.001**	0.669	0.444
N × M	**0.032**	—	—	**0.023**
A × M	**0.010**	—	—	**0.010**

*In the models for Experiment 1, pot was included as the random effect. In the models for Experiment 2, both clonal fragment and pot were included as the random effects; maternal size was included assuming that the growth of ramets was also a function of maternal size. “—” indicates that the interaction term, due to its non-significance and based on Akaike information criterion test, was excluded in the selected model. P-values (in bold when P < 0.05) are given, calculated from F statistics of type 3 hypothesis with the Kenward-Roger’s approximations for the degrees of freedom.*

**Table 2:** Summary of statistics for analyzing the effects of severance treatment in **(A,B)** Experiment 1 and neighbor treatment in **(C,D)** Experiment 2 on root mass fraction (RMF) as a function of ramet total mass, and on leaf and new stolon mass as a function of root mass of *P. reptans* ramets, using linear mixed-effect models.

**Experiment 1: The effects of severing connections**					**Experiment 2: Physiological self/non-self recognition**			
**(A)**	**Fixed effect**	**RMF**			**(C)**	**Fixed effect**	**RMF**	
		
	Severance (S)	0.798				Neighbor (N)	**0.002**	
	Age	**0.002**				Age	0.616	
	Total mass (T)	0.107				Total mass (T)	**<0.001**	
	S × T	—				N × T	**0.002**	
		
**(B)**	**Fixed effect**	**Leaf mass**	**New stolon mass**		**(D)**	**Fixed effect**	**Leaf mass**	**New stolon mass**
		
	Severance (S)	0.481	0.134			Neighbor (N)	0.592	0.272
	Age (A)	0.378	0.106			Age (A)	**<0.001**	**<0.001**
	Root mass (R)	**<0.001**	**<0.001**			Root mass (R)	**<0.001**	**<0.001**
	S × R	—	**0.047**			N × R	—	—
	A × R	—	—			A × R	—	**0.019**



*Pot was included as the random effect in the models for Experiment 1. Clonal fragment and pot were included as the random effects in the models for Experiment 2. “—” indicates that the interaction term, due to its non-significance and based on Akaike information criterion test, was excluded in the selected model. *P*-values (in bold when P < 0.05) are given, calculated from F statistics of type 3 hypothesis with the Kenward-Roger’s approximations for the degrees of freedom.*

### Experiment 2: Physiological Self/Non-Self Recognition

Neighbor treatment had no effect on total mass (*P*= 0.442) or maternal size (*P*= 0.493) of clonal fragments. It had no effect on root mass of younger or older ramets either (**Table 1B**; **Figure 2B**). However, the negative relationship between RMF and total mass of ramets was significantly affected by the disconnected-neighbor treatment (**Table 2C**; **Figure 3C**), leading to significantly higher RMF in that treatment as compared to the closely connected- (*P* = 0.001) or remotely connected-neighbor treatment (*P* = 0.031).

**FIGURE 4:**
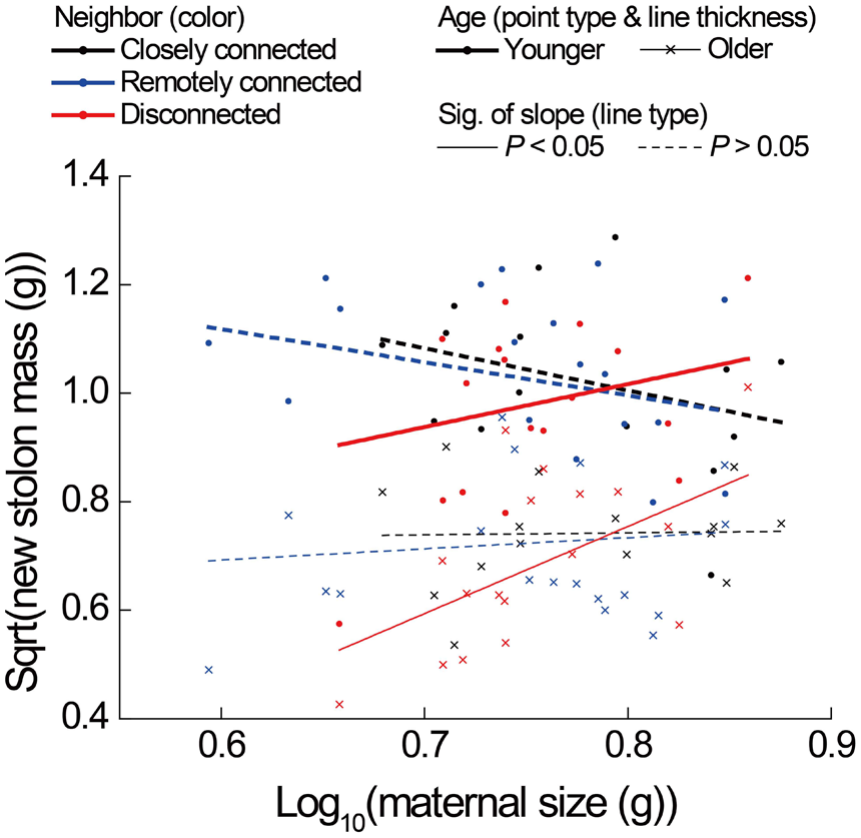
**Effects of neighbor treatment on new stolon mass as a function of maternal size of younger and older *P. reptans* ramets in Experiment 2.** All regression lines are based on the analyses using linear mixed-effect models (see**Table 1B**).

The correlations between new stolon mass and root mass of both younger and older ramets did not differ among three neighbor treatments (**Table 2D**;**Figure 3D**). However, only in the disconnected-neighbor treatment was new stolon mass of both younger and older ramets dependent on their maternal size. In the other two treatments, new stolon mass remained high regardless of maternal size (**Figure 4**, significant neighbor × maternal size in **Table 1B**). This led to a significantly negative overall effect of the disconnected-neighbor treatment on new stolon mass (**Table 1B**) as compared to the closely connected- (*P* = 0.021) or remotely connected-neighbor treatment (*P* = 0.016). Notably, neighbor treatment effects were more profound on younger ramets than on older ones (**Figure 2B**); younger ramets performed much better than older ones in all growth measures (**Table 1B**; **Figure 2**).

## Discussion

In Experiment 1 we show that severing connections generally reduced new stolon mass, but had no effect on root allocation of ramets. However, it did reduce root mass of younger ramets of the pairs. In Experiment 2 we show that the disconnected-neighbor treatment generally reduced new stolon mass, had no effect on root mass but enhanced root allocation of ramets. There was no difference in ramet growth between closely connected- and remotely connected-neighbor treatments. Below, we discuss to what extent our three hypotheses are supported by these results, and whether our novel setup can provide unbiased evidence for physiological self/non-self recognition in clonal plants.

### Effects of Severing Connections

Since the severance of connections reduced new stolon mass of ramets (Experiment 1) to the same extent as the disconnected-neighbor treatment did (Experiment 2), one may argue that results from an “intact/severed pair” setup can provide evidence for physiological self/non-self recognition. However, our analyses reveal that the underlying mechanisms of the two processes are different. That is, the former was caused by a disruption of stolon-root growth relationship (**Figure 3B**), while the latter was caused by a limitation of maternal size (**Figure 4**) and with a consequence of significantly higher root allocation. Although both severance treatment (Experiment 1) and disconnected-neighbor treatment (Experiment 2) had no overall effect on root production (also see *Glechoma hederacea* and *Fragaria vesca*, Semchenko et al., 2007), the former specifically reduced root mass of younger ramets, which did not occur in the latter. Therefore, our results suggest that the growth responses of ramets to the severance effects in an “intact/severed pair” setup do not represent evidence for physiological self/non-self recognition, instead, are more likely to be the consequence of a disruption of physiological integration (e.g., Birch and Hutchings, 1999; van Kleunen and Stuefer, 1999). However, it should be noted that ramets in Experiment 2 remained connected to mother ramets, while this is not the case for ramets in Experiment 1. Thus, the differences may also partly be due to an effect of mother ramets in Experiment 2.

As predicted by our first hypothesis, negative effects of severance on biomass production mainly occurred in younger ramets (**Figure 2A**). This might reflect that as physiological integration mainly entails acropetal movement of resources (e.g., Price and Hutchings, 1992; D’Hertefeldt and Jónsdóttir, 1999), younger ramets would suffer from the loss of source support from the older ramets. This could be further aggravated through competition effects. One may expect that younger ramets in the severed treatment should enhance allocation to roots for resource foraging. However, our results show that severance had no effect on root allocation of younger ramets. This might indicate that the demands for the production of assimilates are also important for them, leading to no reduction of allocation to aboveground parts. On the other hand, severance did not increase biomass of older ramets (**Figure 2A**), indicating that partial support to a younger ramet did not limit the growth of the older (Alpert and Mooney, 1986). This could potentially be explained by the finding that photosynthetic activities of older ramets can be enhanced by the sink demands from their connected younger ramets (Roiloa and Retuerto, 2005; You et al., 2014). Furthermore, total mass of younger ramets was similar to that of older ones in the intact pairs in Experiment 1 (**Figure 2A**), but was remarkably greater in the pairs with closely connected-neighbor treatment in Experiment 2 (**Figure 2B**). This difference might reflect the effect of connectedness to mother ramets, e.g., the source–sink relationships at the level of physiological integration: when a pair of ramets is separated from their clonal fragment (the intact pair in Experiment 1), the older ramet is the source, and the younger is the sink. When the pair is still attached to a clonal fragment (the closely connected pair in Experiment 2), both older and younger ones act more as sinks with their mother ramet being the major source, but the sink of younger ones is probably stronger (Price et al., 1992). Together, it indicates that results of studies using an “intact/severed pair” setup could be potentially confounded by additional effects that are not related to the loss of self-recognition.

### Evidence for Physiological Self/Non-Self Recognition

In contrast to our second and third hypotheses that ramets should produce greater root mass when they interact with disconnected or remotely connected neighbors than with closely connected neighbors, results of our Experiment 2 clearly showed that root production was not affected by any type of neighbors, but was mainly determined by ramet age and maternal size within the clonal system. Interestingly, however, we did find a significant reduction of new stolon mass of ramets in response to the disconnected-neighbor treatment, i.e., only when interacting with disconnected neighbors was new stolon mass of ramets limited by their maternal size (**Figure 4**). As new stolon mass was reduced while root production was more or less maintained, relative allocation to roots increased. These results indicate that clonal fragments are able to adjust their investment strategy of attached ramets based on the physiological identity (i.e., disconnected versus connected) of their belowground neighbors. This provides unbiased evidence for physiological self/non-self recognition, i.e., physical disconnection can disrupt self-recognition within the genet of *P. reptans* we tested. Since there was no significant difference in any measure of ramet growth between closely connected- and remotely connected-neighbor treatments, we found no evidence for a distance effect. This suggests that self-recognition can be maintained over quite a long distance (more than 100 cm in the disconnected-neighbor treatment, B. J. W. Chen pers. obs.) within the clone of this *P. reptans* genotype, contrary to what Gruntman and Novoplansky (2004) concluded for *B. dactyloides*.

So far, the underlying mechanism for this type of self/non-self recognition is still unclear. Although allogeneic recognition involving genetically dependent root exudates has been well investigated in plant-plant interactions between genotypes (e.g., Biedrzycki et al., 2010; Fang et al., 2013; Semchenko et al., 2014) and species (e.g., Badri et al., 2012; Padilla et al., 2013; Schmid et al., 2013), to what extent it also plays a role in the process of physiological self/non-self recognition is in doubt, since genetically identical plants would be expected to produce the same biochemical substances (Chen et al., 2012). A non-allogeneic mechanism has been proposed, i.e., an oscillatory signaling system that relies on physical connection (see Falik et al., 2003; Gruntman and Novoplansky, 2004). However, this still remains untested.

Evolutionary game-theoretical models predict that an optimal plant population with maximum reproduction can be invaded by individuals that grow more leaves, are taller or produce more roots. As a consequence, a population with an evolutionarily stable strategy (i.e., the population cannot be invaded by individuals employing a different growth strategy) is less than maximally reproductive (Anten and During, 2011). This indicates that natural selection favors plants enhancing root allocation for resource competition even at the expense of reproduction in the presence of belowground neighbors (Gersani et al., 2001; O’Brien et al., 2007). It may explain our findings of higher RMF and lower new stolon mass in ramets grown with disconnected (physiological non-self) neighbors. Therefore, our study clearly demonstrates the reproductive benefits from physiological self-recognition. It indicates that such a process may work across large distances within the clones, and thus could contribute to the performance of clonal plants at least in terms of vegetative propagation when the connections remain intact. Our study also suggests that loss of connection may lead to a loss of at least part of the ability to recognize genetically identical units, and this may lead to reduction in clonal propagation. Self-recognition, therefore, may affect the success of clonal plants in many ways and should be included in the study of, e.g., patch expansion (Herben and Novoplansky, 2008), environmental heterogeneity adaptation (van Kleunen and Fischer, 2001) and new habitat invasion (Yu et al., 2009).

As noted, physiological self-recognition has to be shown genotype-dependent in other species (e.g., *F. chiloensis*, Holzapfel and Alpert, 2003; but see *Trifolium repens*, Falik et al., 2006). The current study was conducted with a single genotype. The extent to which our results can be generalized for the species *P. reptans* is still unknown. Therefore, we suggest that future studies in this direction should use multiple genotypes.

### A Caution of Rooting Volume

Finally, a potential rooting volume effect should be considered (Hess and de Kroon, 2007; Murphy et al., 2013). In our Experiment 2 each clonal fragment had two pots (in addition to the pot for the mother ramet) for four daughter ramets growing roots in closely connected- and remotely connected-neighbor treatments but four pots in the disconnected-neighbor treatment to exploit the same amount of resources. Plants commonly produce more roots in larger rooting volumes (reviewed in Poorter et al., 2012) which could be at the expense of investments to other organs (Hess and de Kroon, 2007). If this rooting volume effect would have been dominant in our study, it would have likely led to greater overall root production of clonal fragments in disconnected-neighbor treatment than in closely connected- or remotely connected-neighbor treatments. This is, however, contrary to the results of non-significant neighbor treatment effect (*F* = 0.041, *P* = 0.959). Therefore, disconnection effect in Experiment 2 is unlikely to be associated with the difference in total rooting volumes of clonal fragments.

## Conclusion

This study provides a novel setup to test for disconnection and distance effects on physiological self/non-self recognition in clonal plants without severing connections between target ramets. By contrasting the growth responses of *P. reptans* ramets in Experiment 1 using a traditional “intact/severed pair” setup and Experiment 2 using our new setup, we clearly show that a sudden severance of connections on ramet growth acts as a disruption of physiological integration rather than as a loss of self-recognition. Results from our Experiment 2 provide unbiased evidence for physiological self/non-self recognition, and suggest that such self-recognition can persist over a relatively long distance within a clone of the *P. reptans* genotype we used. Since clonal growth is widespread in the plant kingdom (Klimeš et al., 1997), physiological self/non-self recognition may potentially play an important role in the network of interactions within plant communities (Gruntman and Novoplansky, 2004). For a better understanding of its evolutionary and ecological impacts, the next important steps are to investigate the underlying mechanisms and assess its generality within and among species.

## Conflict of Interest Statement:

The authors declare that the research was conducted in the absence of any commercial or financial relationships that could be construed as a potential conflict of interest.
